# The effectiveness of manual therapy for the management of musculoskeletal disorders of the upper and lower extremities: a systematic review by the Ontario Protocol for Traffic Injury Management (OPTIMa) Collaboration

**DOI:** 10.1186/s12998-015-0075-6

**Published:** 2015-10-27

**Authors:** Danielle Southerst, Hainan Yu, Kristi Randhawa, Pierre Côté, Kevin D’Angelo, Heather M. Shearer, Jessica J. Wong, Deborah Sutton, Sharanya Varatharajan, Rachel Goldgrub, Sarah Dion, Jocelyn Cox, Roger Menta, Courtney K. Brown, Paula J. Stern, Maja Stupar, Linda J. Carroll, Anne Taylor-Vaisey

**Affiliations:** Rebecca MacDonald Centre for Arthritis and Autoimmune Disease, Mount Sinai Hospital, 600 University Avenue, Toronto, Ontario Canada M5G 1X5; UOIT-CMCC Centre for the Study of Disability Prevention and Rehabilitation, University of Ontario Institute of Technology (UOIT) and Canadian Memorial Chiropractic College (CMCC), 6100 Leslie St, Toronto, Ontario Canada M2H 3J1; Graduate Education and Research Programs, Canadian Memorial Chiropractic College (CMCC), 6100 Leslie St, Toronto, Ontario Canada M2H 3J1; Division of Undergraduate Education, Canadian Memorial Chiropractic College (CMCC), 6100 Leslie St, Toronto, Ontario Canada M2H 3J1; Canada Research Chair in Disability Prevention and Rehabilitation, University of Ontario Institute of Technology (UOIT), 2000 Simcoe St N, Science building, Room 3000, Oshawa, Ontario Canada L1H 7K4; Faculty of Health Sciences, University of Ontario Institute of Technology (UOIT), 2000 Simcoe St N, Science building, Room 3000, Oshawa, Ontario Canada L1H 7K4; UOIT-CMCC Centre for the Study of Disability Prevention and Rehabilitation, 6100 Leslie St, Toronto, Ontario Canada M2H 3J1; Department of Graduate Studies, Canadian Memorial Chiropractic College (CMCC), 6100 Leslie St, Toronto, Ontario Canada M2H 3J1; Graduate Education Program, Canadian Memorial Chiropractic College (CMCC), 6100 Leslie St, Toronto, Ontario Canada M2H 3J1; Graduate Education and Research, Canadian Memorial Chiropractic College (CMCC), 6100 Leslie St, Toronto, Ontario Canada M2H 3J1; Injury Prevention Centre and School of Public Health, University of Alberta, 4075 Research Transition Facility, 8308-114 St, Edmonton, Alberta Canada T6G 2E1

**Keywords:** Manual therapy, Musculoskeletal disorders, Upper and lower extremities, Treatment, Rehabilitation, Recovery, Outcome, Systematic review

## Abstract

**Background:**

Musculoskeletal disorders (MSDs) of the upper and lower extremities are common in the general population and place a significant burden on the health care system. Manual therapy is recommended by clinical practice guidelines for the management of these injuries; however, there is limited evidence to support its effectiveness. The purpose of our review was to investigate the effectiveness of manual therapy in adults or children with MSDs of the upper or lower extremity.

**Methods:**

Randomized controlled trials (RCTs), cohort studies, and case–control studies evaluating the effectiveness of manual therapy were eligible. We searched MEDLINE, EMBASE, PsycINFO, CINAHL, and the Cochrane Central Register of Controlled Trials from 1990 to 2015. Paired reviewers screened studies for relevance and critically appraised relevant studies using the Scottish Intercollegiate Guidelines Network criteria. Studies with low risk of bias were synthesized following best-evidence synthesis principles. Where available, we computed mean changes between groups, relative risks and 95 % CI.

**Results:**

We screened 6047 articles. Seven RCTs were critically appraised and three had low risk of bias. For adults with nonspecific shoulder pain of variable duration, cervicothoracic spinal manipulation and mobilization in addition to usual care may improve self-perceived recovery compared to usual care alone. For adults with subacromial impingement syndrome of variable duration, neck mobilization in addition to a multimodal shoulder program of care provides no added benefit. Finally, for adults with grade I-II ankle sprains of variable duration, lower extremity mobilization in addition to home exercise and advice provides greater short-term improvements in activities and function over home exercise and advice alone. No studies were included that evaluated the effectiveness of manual therapy in children or for the management of other extremity injuries in adults.

**Conclusions:**

The current evidence on the effectiveness of manual therapy for MSDs of the upper and lower extremities is limited. The available evidence supports the use of manual therapy for non-specific shoulder pain and ankle sprains, but not for subacromial impingement syndrome in adults. Future research is needed to determine the effectiveness of manual therapy and guide clinical practice.

**Systematic review registration number:**

CRD42014009899

## Background

Musculoskeletal disorders (MSDs) of the upper and lower extremities are common. In the United States, 36 % and 16 % of injuries presenting to emergency departments are sprains and/or strains of the lower and upper extremities, respectively [1, 2]. In Canada, more than 75 % of individuals injured in a motor vehicle collision report upper extremity pain and 27.5 % report pain in the lower extremity [3]. In Dutch adults, the point prevalence of upper and lower extremity pain are 41 % (i.e., shoulder, elbow and writs/hand pain) and 20 % (i.e., knee and ankle pain), respectively [4].

Injuries of the upper and lower extremities represent a significant portion of the burden of MSDs in the workplace. In 2013 in the United States, the median number of days away from work for upper and lower extremity injuries were 10 and 12 days respectively with shoulder and knee injuries, accounting for the largest number of lost work days [5]. In 2014 in Ontario, 22.4 % and 19.3 % of all workers’ approved lost time compensation claims are related to upper extremity injuries and lower extremity injuries, respectively [6].

Patients frequently seek manual therapy including manipulation, mobilization, and traction for the management of MSDs of extremities [7–9]. Manual therapy is often recommended as a component of rehabilitation programs for the management of MSDs of extremities [10–12]. For example, the Workplace Safety and Insurance Board (WSIB) of Ontario recommends the use of manipulation and/or mobilization for the management of MSDs of the extremities [10]. Similarly, manual therapy is recommended in practice guidelines for the management of rotator cuff syndrome in Australia [11]. In 2009, the Council for Chiropractic Guidelines and Practice Parameters (CCGPP) recommended the use of manipulative therapy for the management of lower extremity injuries [12]. However, these recommendations need to be updated (i.e., published earlier than in the past five years) [10–12]. Previous systematic reviews reported inconsistent results on the effectiveness of manual therapy for the management of MSDs [13–21]. This can be attributed to the publication of new evidence and differences in methodology (e.g., incomprehensive search strategy, including small sample trials).

Therefore, an up-to-date systematic review is needed to evaluate the effectiveness of manual therapy for the treatment of MSDs of the extremities. The purpose of our systematic review was to investigate the effectiveness of manual therapy compared to other interventions, placebo/sham interventions or no intervention in improving self-rated recovery, functional recovery (e.g., return to activities, work or school), or clinical outcomes (e.g., pain, health-related quality of life, depression) in patients with MSDs of the upper or lower extremity.

## Methods

### Registration

This systematic review protocol was registered with the International Prospective Register of Systematic Reviews (PROSPERO) on May 21, 2014 (CRD42014009899).

### Searches

We developed our search strategy with a health sciences librarian (Appendix 1 and 2). A second librarian reviewed the search strategy for completeness and accuracy using the Peer Review of Electronic Search Strategies (PRESS) Checklist [22, 23]. We searched the following databases: MEDLINE, EMBASE, CINAHL, PsycINFO, and the Cochrane Central Register of Controlled Trials, from January 1, 1990 to April 8, 2015 for studies related to the lower extremity and from January 1, 1990 to April 14, 2015 for studies related to the upper extremity. As a supplement, we hand-searched the reference lists of previous systematic reviews for any additional relevant studies.

The search strategies were first developed in MEDLINE and subsequently adapted to the other bibliographic databases. Search terms included combined controlled vocabulary specific to each database (e.g. Medical Subject Headings [MeSH] for MEDLINE) and text words relevant to our research question and selection criteria. We used EndNote X7 to create a bibliographic database to manage search results.

### Study inclusion and exclusion criteria

#### Population

Our review targeted studies of adults (18 years and older) and/or children with MSDs of the upper or lower extremity. We defined MSDs, based on the Centers for Disease Control and Prevention (CDC) definition, as grade I-II sprain/strains, tendinitis, tendinosis, tendinopathy, nonspecific pain of the upper extremity (i.e., shoulder, elbow, forearm, wrist, hand) or lower extremity (i.e., hip, thigh, knee, leg, ankle, foot), or other MSDs (including neuropathies) as informed by available evidence [24]. Specific diagnoses considered for the upper extremity included but not limited to: subacromial impingement, olecranon bursitis, lateral epicondylitis, medial epicondylitis, cubital tunnel syndrome, carpal tunnel syndrome, and De Quervain’s tenosynovitis. In the lower extremity, we considered specific diagnoses including but not limited to: patellofemoral pain (syndrome), iliotibial band syndrome, Achilles tendinopathy, and plantar fasciitis. We defined the grades of sprains and strains according to the classification proposed by the American Academy of Orthopaedic Surgeons (Tables 1 and 2) [25]. We excluded studies involving major pathology (e.g., fractures, dislocations, infection, neoplasms, or systemic disease). Studies of grade I-III ankle sprains and strains were considered if a grade specific analysis was conducted or if a trial included the same distribution of grade III injuries between intervention groups. Studies including other grades of sprains or strains in the upper or lower extremity had to provide separate results for participants with grade I and/or II sprains/strains.

**Table 1:** Case definition of sprains [25]

Grade	Definition
I	Sprain occurs when ligamentous fibers are stretched but remain structurally intact.
II	Sprain occurs when ligamentous fibers become partially torn. Physical stress reveals increased laxity with a definite end point.
III^a^	Sprain occurs when a ligament is completely torn, leading to gross instability.

^a^Grade III sprains are excluded from this review; grade I-III ankle sprains and strains were considered if a grade specific analysis was conducted or if a trial included the same distribution of grade III injuries between intervention groups

**Table 2:** Case definition of strains [25]

Grade	Definition
I	Strain occurs when less than 5 % of muscle/fibers are disrupted, with fascia remaining intact.
II	Strain occurs when muscles fibers/tendon discontinuity involves a moderate number of muscle fibers.
III^a^	Strain occurs when there is complete discontinuity in the muscle fibers.

^a^Grade III strains are excluded from this review; grade I-III ankle sprains and strains were considered if a grade specific analysis was conducted or if a trial included the same distribution of grade III injuries between intervention groups

#### Interventions

We restricted our review to studies that tested the effectiveness of manual therapy. We defined manual therapy as techniques that involve the application of hands-on and/or mechanically assisted treatments to the spine or joints of the upper and lower extremities including manipulation, mobilization and traction but excluding soft tissue therapy. Specifically, mobilization includes techniques incorporating a low velocity and small or large amplitude oscillatory movement, within a joint’s passive range of motion [26, 27]. Manipulation includes techniques incorporating a high velocity, low amplitude impulse or thrust applied at or near the end of a joint’s passive range of motion [27]. Manual or mechanically assisted traction is defined as a manual or mechanically assisted application of an intermittent or continuous distractive force [28, 29].

#### Comparison groups

We included studies that compared one or more manual therapy interventions to one another or one manual therapy intervention to other modes of interventions, wait list, placebo/sham interventions, or no intervention.

#### Outcomes

To be eligible, studies had to include one of the following outcomes: 1) self-rated recovery (e.g., self-reported on a Likert Scale); 2) functional recovery (e.g., measured with the Foot and Ankle Ability Measure [FAAM], the Lower Extremity Functional Scale [LEFS], the Quick Disabilities of the Arm, Hand, and Shoulder [QuickDASH]); 3) pain intensity (e.g., measured with Numerical Rating Scale [NRS]); 4) health-related quality of life (e.g., measured with EuroQol); or 5) adverse events.

#### Study characteristics

Eligible studies met the following criteria: 1) English language; 2) randomized controlled trials (RCTs), cohort studies, or case–control studies; 3) included an inception cohort of a minimum of 30 participants per treatment arm with the specified condition for RCTs or 100 participants per group with the specified condition in cohort studies or case–control studies. In RCTs, a sample size of 30 is conventionally considered the minimum needed for non-normal distributions to approximate the normal distribution [30]. The assumption that data is normally distributed is required to ascertain a difference in sample means between treatment arms. A research finding is also less likely to be true due to smaller power when the studies conducted in a field have smaller sample sizes [31]. Furthermore, small sample sizes increase the risk of residual confounding [32–34].

We excluded studies with the following characteristics: 1) publication types including letters, editorials, commentaries, unpublished manuscripts, dissertations, government reports, books and book chapters, conference proceedings, meeting abstracts, lectures and addresses, consensus development statements, or guideline statements; 2) study designs including pilot studies, cross-sectional studies, case reports, case series, qualitative studies, narrative reviews, systematic reviews (with or without meta-analyses), clinical practice guidelines, biomechanical studies, laboratory studies, and studies not reporting on methodology; 3) cadaveric or animal studies.

### Study selection

We used a two-phase screening process to select eligible studies. In phase one, paired reviewers screened citation titles and abstracts to determine the eligibility of studies. Phase one screening resulted in studies being classified as relevant, possibly relevant or irrelevant. Studies were classified as relevant if all inclusion criteria were met. Studies that did not meet any one of our inclusion criterion or had met any one of the exclusion criteria were deemed irrelevant. Studies where insufficient information was provided in their titles and abstracts to not allow the determination of eligibility were classified as possibly relevant. Possibly relevant studies entered a phase two screening. In phase two, the same pairs of reviewers independently screened the full text of possibly relevant studies to determine eligibility using the same inclusion and exclusion criteria as in phase one. Reviewers met to resolve disagreements and reach consensus on the eligibility of studies. A third reviewer was used if consensus could not be reached.

### Assessment of risk of bias

Paired reviewers critically appraised the internal validity of eligible studies using the Scottish Intercollegiate Guidelines Network (SIGN) criteria [35]. The SIGN criteria were used to qualitatively evaluate the presence and impact of selection bias, information bias, and confounding on the results of a study. We did not use a quantitative score or a cut-off point to determine the internal validity of studies [36]. Rather, the SIGN criteria were used to assist reviewers make an informed overall judgment on the internal validity of studies. This methodology has been previously described [37–42].

Specifically, we critically appraised the following methodological aspects of each study: 1) clarity of the research question; 2) randomization method; 3) concealment of treatment allocation; 4) blinding of treatment and outcomes; 5) similarity of baseline characteristics between/among treatment arms; 6) co-intervention contamination; 7) validity and reliability of outcome measures; 8) follow-up rates; 9) analysis according to intention-to-treat principles; and 10) comparability of results across study sites (where applicable). Reviewers reached consensus through discussion. An independent third reviewer resolved disagreements if consensus could not be reached. Authors were contacted when additional information was needed to complete the critical appraisal. Studies with adequate internal validity (i.e. low risk of bias) were included in our evidence synthesis [43].

### Data extraction and synthesis of results

The lead author extracted data from scientifically admissible studies into evidence tables (Table 4). A second reviewer independently checked the extracted data. The evidence tables included key information of each study (i.e., author(s), year; subjects and setting; interventions; comparisons; follow-up period; outcomes measured; key findings). Meta-analysis was not performed due to heterogeneity of scientifically admissible studies with respect to patient populations, interventions, comparators and outcomes. We performed a qualitative synthesis of findings from scientifically admissible studies to develop evidence statements according to principles of best-evidence synthesis [43].

We used standardized measures (i.e., minimal clinically important differences [MCIDs]) to determine the clinical importance of changes reported in each trial for common outcome measures. These include a between-group difference of 2.5/10 on the Numeric Rating Scale (NRS) [44]; 11.2/100 on the Quick Disabilities of the Arm, Hand, and Shoulder (QuickDASH) [44]; 8/100 on the Foot and Ankle Ability Measure (FAAM) activities of daily living (ADL) subscale [45], 9/100 on the FAAM sport subscale [45], and 9/80 on the Lower Extremity Functional Scale (LEFS) [46]. The MCID for the Shoulder Disability Questionnaire (SDQ) is not known.

### Statistical analyses

We computed agreement between reviewers for the screening of articles and reported the kappa statistic (K) and 95 % confidence interval (CI) [47]. Where available, we used data provided in the admissible articles to measure the association between the tested interventions and the outcomes by computing the relative risk (RR) and its 95 % CI (e.g., self-perceived recovery, recurrence or satisfaction). Similarly, we computed differences in mean changes between groups and 95 % CI to quantify the effectiveness of interventions. The calculation of 95 % CIs was based on the assumption that baseline and follow-up outcomes were highly correlated (r = 0.80) [48, 49].

### Reporting

This systematic review was organized and reported based on Preferred Reporting Items for Systematic Reviews and Meta-Analyses (PRISMA) statement [50].

## Results

### Study selection

**Fig. 1:**
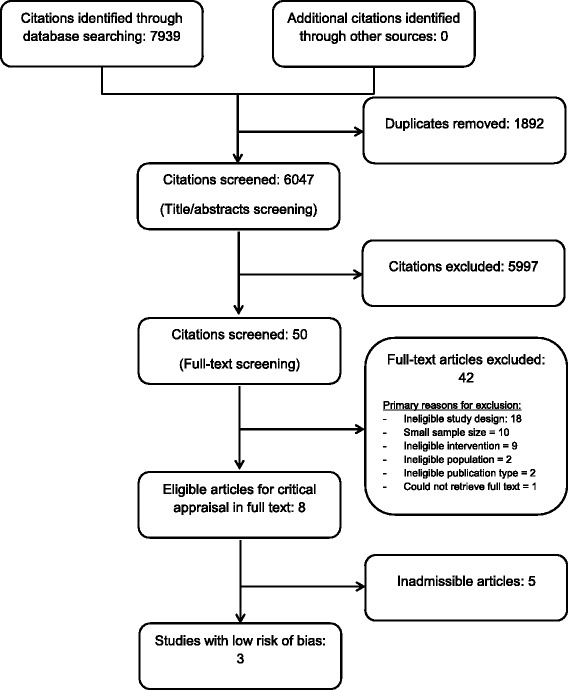
Identification and Selection of Articles

We screened 6047 titles and abstracts, of which eight articles (reporting results from seven RCTs) were eligible for critical appraisal [51–58] (Fig. 1). Three RCTs had low risk of bias and were included in our synthesis [51–53]. The inter-rater aggreement for article screening was k = 0.89 (95 % CI 0.74–1.00) for articles related to the upper extremity; and k = 1.0 for articles related to the lower extremity, but this is best described as a kappa paradox caused by a low prevalence of relevant studies [59]. The percent agreement for the critical appraisal of studies was 71.4 % (5/7 studies). Disagreement was resolved through discussion. We contacted authors from two studies [52, 56] during critical apprasial to request additional information; no authors responded.

### Study characteristics

All three studies examined the effectiveness of manual therapy in adults [51–53]. One RCT examined the effectiveness of spinal manual therapy (i.e., manipulation and mobilization to the cervical spine, upper thoracic spine, and adjacent ribs) for the management of nonspecific shoulder pain of variable duration [51]; one RCT examined the effectiveness of prone grade III posterior-anterior mobilization of the cervical spine for the management of subacromial impingement syndrome of variable duration [52]; and one RCT examined the effectiveness of lower extremity mobilization for the management of grade I-II inversion ankle sprains [53] (Table 3).

**Table 3:** Summary of assessment of risk of bias for accepted randomized controlled trials based on Scottish Intercollegiate Guidelines Network (SIGN) criteria [35]

Author, Year	Research Question	Random-ization	Conceal-ment	Blinding	Similarity at baseline	Similarity between arms	Outcome measure-ment	Percent drop-out^a^	Intention to treat	Results comparable between sites
Bergman et al., 2004 [40]	Y	CS	Y	Y	N^b^	Y	Y	12 weeks (immediately post-intervention)	Y	CS
								UC = 14 %		
								MT + UC = 13 %		
								26 weeks:		
								UC = 11 %		
								UC + MT = 9 %		
								52 weeks:		
								UC = 13 %		
								MT + UC = 6 %		
Cook et al., 2014 [41]	Y	Y	N	Y	Y	CS	Y	Post-intervention:	N	CS
								Manipulation: 2/38 = 5 %		
								Control: 4/36 = 11 %		
Cleland et al., 2013 [42]	Y	Y	Y	Y	Y	N	Y	1 month:	Y	CS
								MTEX: 3/37 = 8 %		
								HEP: 2/37 = 5 %		

Acronyms: *Y* Yes, *N* No, *CS* Can’t Say, *NA* Not Applicable, *MT* Manual therapy, *UC* Usual Care, *MTEX* manual therapy and home exercise, *HEP* Home exercise program

^a^Percent drop-out includes drop-outs and loss to follow-up

^b^Baseline differences were adjusted in the analysis

### Risk of bias within studies

All three studies with low risk of bias clearly stated their research questions, implemented appropriate blinding of outcome measurement, and used valid and reliable outcome measures (Table 1). All studies had follow-up rates greater than 80 %. However, these studies had the following limitations: 1) unclear methods of randomization (1/3) [51]; 2) unclear methods of allocation concealment (1/3) [52]; 3) clinically important differences between treatment groups at baseline that were adjusted in the analysis (1/3) [51], 4) limited information or differences between groups with respect to co-intervention (2/3) [52, 53]; and 5) failure to use an intention-to-treat analysis (1/3) [52].

Four RCTs had high risk of bias and were excluded from the synthesis. These studies had the following limitations: 1) poor or unknown randomization methods (2/4) [54, 57, 58]; 2) poor or unknown allocation concealment methods (3/4) [54, 56–58]; 3) outcome assessor not blinded or blinding status not clear (3/4) [54–56]; 4) clinically important differences in baseline characteristics (2/4) [55, 57, 58]; 5) drop-outs not reported or large differences in drop-out rates between treatment arms (4/4) [54–58]; 6) use of outcome measures that were not valid or reliable (1/4) [57, 58]; 7) no mention of the use of an intention-to-treat analysis (1/4) [56]; 8) limited information or differences between groups with respect to co-interventions [54, 56–58].

### Summary of evidence

#### Nonspecific shoulder pain of variable duration

Evidence from one RCT suggests that adding manual therapy (i.e., spinal manipulation and mobilization) to usual care may improve self-perceived recovery compared to usual care alone for the management of nonspecific shoulder pain and dysfunction of the cervicothoracic spine [51]. In a study by Bergman et al., adults with nonspecific shoulder pain and dysfunction (i.e. pain or restricted movement) in the cervicothoracic spine and adjacent ribs were allocated to either manual therapy combined with usual care or usual care alone. Paritcipants in the manual therapy group received up to six sessions over 12 weeks of manipulation and mobilization to the cervical spine, upper thoracic spine, and adjacent ribs by a physiotherapist. Usual care was provided as outlined by the Dutch College of General Practitioners and could involve information, advice, medication, corticosteroid injections, and physiotherapy. Participants in the manual therapy group were more likely to report ‘completely recovered’ or ‘much improved’ immediately following the 12 weeks intervention [RR 2.0 (95 % CI 1.2, 3.4)] and at the 52 weeks follow-up [RR 1.5 (95 % 1.0, 2.2)] but not at the 26 weeks follow-up (Table 4). Furthermore, the manual therapy group was more likely to report their symptoms to be improved to the point where they were no longer inconvenient at the 52 weeks follow-up [RR 1.4 (95 % CI 1.0, 1.9)]. There were statistically significant but not clinically important differences favouring the manual therapy group for pain (NRS) at the 12, 26, and 52 weeks follow-ups. Moreover, there were statistically significant differences favouring the manual therapy group for disability (SDQ) at the 26 weeks follow-up but not immediately following the 12 weeks intervention or at the 52 weeks follow-up (Table 4). The clinical importance of this finding is not known. There were no important differences between groups in health-related quality of life. Treatment preference may have biased the outcome in favour of the manual therapy group, because 12 % more participants in the usual care group prefered manual therapy at baseline.

**Table 4:** Evidence table for accepted randomized controlled trials assessing the effectiveness of manual therapy for musculoskeletal disorders of the upper and lower extremities

Author(s), Year	Subjects and Setting; Number (n) Enrolled	Interventions; Number (n) of Subjects	Comparisons; Number (n) of Subjects	Follow-up	Outcomes	Key Findings
Bergman et al., 2004 [51]	Participants (=18 y.o) recruited from general practices in Groningen, the Netherlands.	Manual therapy and usual care:	Usual care:	12 (immediately post-intervention), 26 and 52 weeks	Primary outcomes:	Patient-perceived recovery (manual therapy and usual care vs. usual care):
	Case definition: Pain of variable duration between the neck and elbow at rest or during movement of the upper arm; physical examination confirming shoulder symptoms and dysfunction in the cervicothoracic spine and ribs with accompanying pain or restricted movement (n=150)	Manual therapy (up to 6 sessions over 12 weeks) by physiotherapists: manipulations and mobilization to the cervical spine, upper thoracic spine, and adjacent ribs.	Usual care (information, advice, and therapy) as outlined by the Dutch College of General		Self-perceived recovery (7point Likert scale; recovered = “completely recovered” or “very much improved”)	Proportion of participants reporting themselves ‘completely recovered’ or ‘verymuch improved’ (reference group: usual care)^a^:
		Usual care (information, advice, and therapy) according to the Dutch College of General	Practitioners provided by GPs: delivered following same protocol as in manual therapy and usual care group (n=71)		Cure rate (self-report of shoulder symptom improvement to a point where they are no longer inconvenient)	12 weeks: RR 2.0 (95% CI 1.2, 3.4)
						26 weeks: RR 1.2 (95% CI 0.8, 1.7)
		Practitioners provided by GPs:				
						52 weeks: RR 1.5 (95% 1.0, 2.2)
		Weeks 1–2: information about the nature and course of shoulder symptoms, advice on daily activities, prescription for oral analgesics or NSAIDs if necessary.				
						Proportion of participants reporting symptom improvement to the point where they are no longer inconvenient^a^:
						12 weeks: RR 1.4 (95% CI 0.9, 2.0)
						26 weeks: RR 1.2 (95% CI 0.9, 1.8)
						52 weeks: RR 1.4 (95% CI 1.0, 1.9)
						Difference in mean change (manual therapy and usual care – usual care):
						Severity of main complaint (0–10)
						12 weeks: 1.5 (95% CI 0.5, 2.5)
						26 weeks: 1.2 (95% CI 0.2, 2.2)
						52 weeks: 1.4 (95% CI 0.4, 2.4)
						Shoulder disability (0–100)
						12 weeks: 8.5 (95% CI -2.0, 18.9)
						26 weeks: 12.7 (95% CI 1.3, 24.1)
						52 weeks: 6.9 (95% CI -3.5, 20.7)
						General health (3 point scale)
						No difference.
		Weeks 3–4: extension of prescription medication if necessary.			Secondary outcomes: severity of main complaint (NRS, 0–10); functional disability (SDQ, 0–100), quality of life (EuroQol, 5 items scored using 3-point ordinal scale, -1=worst; 1=best).	
		Weeks 5–6: Up to 3 subacromial or glenohumeral corticosteroid injections (40mg triamcinolone acetonide with or without 10mg lidocaine)				
		Weeks 6–12: Physiotherapy shoulder exercises, massage and passive physical modalities were considered. (n=79)				
Cook et al., 2014 [52]	Patients (=18 y.o.) attending outpatient clinical/academic centers in the USA or South Africa.	Shoulder and neck treatment by physiotherapist:	Shoulder treatment by physiotherapist:	Immediately post-intervention [mean 56.1 days (SD 55.0)]	Primary outcome: Disability (QuickDASH, 0–100)	Difference in mean change (shoulder and neck treatment – shoulder treatment) ^a^:
					Secondary outcome: Pain (NRS, 0–10), patient satisfaction and adaptation to symptoms (PASS, acceptable = unlikely to seek further treatment, unacceptable = likely to seek further treatment)	Disability (QuickDASH 0–100):
	Case definition: Shoulder impingement syndrome (mean duration 11.7 weeks) with: 1) pain or dysfunction with overhead activities and active shoulder movements; 2) positive Neer/ Hawkins-Kennedy test; 3) onset =12 months; 4) painful arc; 5) baseline pain =2/10 (n=74)					
		Neck treatment (duration and frequency of treatment determined by the physiotherapist): Grade III posterior-anterior mobilization (3 x 30 oscillations) to stiffest or most painful segments in the cervical spine or to the C5-C6, or C6-C7 segments on the same side of shoulder impingement if joint findings were absent.	Pragmatically delivered multimodal program of care including manual therapy stretching, isotonic strengthening, and restoration of normative movement. (n=36)			
						Post-intervention: 5.3 (95% CI -3.0, 13.6)
						Pain (NRS 0–10)
						Post-intervention: 0.5 (95% CI -0.6, 1.6)
						No difference in the proportion of participants considering their state ‘acceptable’ (unlikely to seek further treatment) ^a^:
						Post-intervention: RR 0.92 (95% CI 0.73, 1.15)
		Multimodal shoulder care: manual therapy, stretching, isotonic strengthening, and restoration of normative movement. (n=38)				No adverse events reported.
Cleland et al., 2013 [53]	Patients (16–60 y.o.) with inversion ankle sprain presenting to physical therapy clinics in Colorado.	MTEX:	HEP:	4 weeks (immediately post-intervention) and 6 months	Primary outcome: Disability (FAAM ADL subscale; 0–100).	Differences in mean change (MTEX-HEP):
		Manual therapy by physical therapist (2 x 30 minute sessions per week for 4 weeks): Grade I-IV mobilization (grade selected by therapist /patient tolerance) to the proximal tibiofibular joint, distal tibiofibular joint, talocrural joint, and subtalar joint.	Home exercises (daily): Instruction by a physical therapist (1 x 30 minute session per week for 4 weeks): same exercises as MTEX group			FAAM ADL (0–100):
		Home exercises (daily): mobilizing exercises for the foot and ankle, gentle strengthening exercises, resistive-band exercises, 1-leg standing activities, standing on balance board, and weight-bearing functional activities; program progressed by physical therapist as indicated				
		Advice to continue with activities that did not increase symptoms and avoid activities that aggravate symptoms.				
		Education on ice, compression, and elevation. (n=37)				
						1 month: 11.7 (95% CI 7.4, 16.1)
						6 months: 6.2 (95% CI 0.98, 11.5)
					Secondary outcomes: Disability (FAAM sports subscale; 0–100); Function (LEFS; 0–80); Pain (NRS; 0–10); global improvement (-7 to +7); recurrence	FAAM sports (0–100):
	Case definition: grade 1 or 2 inversion ankle sprain as defined by the West Point Ankle Sprain Grading System; no restriction in days since injury; NRS = 3/10 in last week; negative Ottawa ankle rules. (n=74)		Advice to continue with activities that did not increase symptoms and avoid activities that aggravate symptoms			1 month: 13.3 (95% CI 8.0, 18.6)
						6 months: 7.2 (95% CI 2.6, 11.8)
						LEFS (0–80):
						1 month: 12.8 (95% CI 9.1, 16.5)
						6 months: 8.1 (95% CI 4.1, 12.1)
			Education on ice, compression, and elevation. (n=37)			NRS (0–10):
						1 month: 1.2 (95% CI 0.9, 1.5)
						6 months: 0.47 (95% CI 0.05, 0.90)
						Global Improvement:
						Statistically significant difference in favor of MTEX at 1 and 6 months (p<0.001).
						Recurrence^a^
						No difference in the proportion of participants reporting recurrence of their injury at 6 months:
						RR 0.6 (95% CI 0.15; 2.33)
						No adverse events were reported.

Acronyms: *ADL* Activities of Daily Living, *FAAM* Foot and Ankle Ability Measure, *GP* General Practitioner, *HEP* Home exercise program, *LEFS* Lower Extremity Functional Scale, *MTEX* Manual therapy and exercise program, *NRS* Numeric Rating Scale, *RR* Relative Risk, *SDQ* Shoulder Disability Questionnaire, *QuickDASH* the Quick Disabilities of the Arm, Shoulder, and Hand

^a^recalculated data from study

#### Subacromial impingement syndrome of variable duration

Evidence from one RCT suggests that adding neck mobilization to a multimodal shoulder program of care does not provide added benefit to patients with shoulder impingement syndrome [52]. In an RCT by Cook et al., adults with subacromial impingement syndrome (mean duration 11.7 weeks) were randomized to a standardized multimodal program of care with or without manual therapy of the cervical spine. The multimodal care included self- and externally applied stretching, isotonic strengthening, and restoration of normative movement. The manual therapy intervention involved prone grade III posterior-anterior mobilization of the cervical spine (30 oscillations repeated three times). Both treatments were delivered by physiotherapists. There were no statistically significant or clinically important differences immediately post-intervention between groups for disability (QuickDASH), pain (NRS) or the proportion of participants considering their state to be acceptable (i.e. no need to seek further intervention) (Table 4).

#### Grade I-II ankle sprains of variable duration

Evidence from one RCT suggests that adding mobilization to home exercise and advice may be more beneficial, in the short-term, than home exercise and advice alone for grade I-II ankle sprains of variable duration [53]. Cleland et al. randomized adults presenting to physical therapy clinics with grade I-II inversion ankle sprains to receive: 1) lower extremity manual therapy combined with home exercises and advice; or 2) home exercise and advice alone [53]. Manual therapy was performed by physical therapists and included grade I-IV mobilization directed at the proximal and distal tibiofibular joints, talocrural joint, and subtalar joint. The grade of mobilization was selected at the discretion of the physical therapist and in consideration of patient tolerance. The home exercise program included progressive daily mobilizing and strengthening exercises (Table 4). There were statistically significant and clinically important differences favouring the manual therapy group immediately following the four weeks intervention for the activities of daily living subscale of the FAAM [mean change difference: 11.7/100 (95 % CI 7.4, 16.1)], the sports subscale of the FAAM [mean change difference: 13.3/100 (95 % CI 8.0, 18.6), and function in LEFS [mean change difference: 12.8/80 (95 % CI 9.1, 16.5)]. There were statistically significant but not clinically important differences favouring the manual therapy group for both FAAM and the LEFS scales at the six month follow-up. There were statistically significant, but not clinically important differences in pain (NRS) favoring the manual therapy group immediately following the four week intervention and at the six month follow-up. Finally, there were no differences in the proportion of participants reporting recurrence of injury at the six month follow-up.

#### Adverse events

Two of the three RCTs with low risk of bias measured adverse events [52, 53]. No adverse events were reported.

## Discussion

### Summary of evidence

Few high-quality studies were available to inform the effectiveness and safety of manual therapy for the management of MSDs of the upper and lower extremities. We identified three studies with low risk of bias that investigated the effectiveness of manual therapy in adults with MSDs of the upper and lower extremities. For nonspecific shoulder pain of variable duration, adding spinal manipulation and mobilization to usual care may improve self-perceived recovery compared to usual care alone. For subacromial impingement syndrome of variable duration, neck mobilization does not provide added benefit when combined with multimodal care. Furthermore, for grade I-II ankle sprains of variable duration, lower extremity mobilization provides added short-term improvements in activities and function when combined with home exercise and advice.

### Previous systematic reviews

Previous systematic reviews reported inconsistent results on the effectiveness of manual therapy for the management of MSDs of the shoulder [16–21]. For the management of nonspecific shoulder pain, our conclusion that manipulation and mobilization may be effective agrees with two previous systematic reviews examining manipulation and mobilization [16] or mobilization [18]; however two other reviews reported inconclusive evidence for the effectiveness of mobilization [17] or manipulation [21]. Our conclusion that neck mobilization does not provide additional benefit to a multimodal program of care for the treatment of subacromial impingement syndrome does not agree with a previous systematic review which found inconclusive evidence for the effectiveness of mobilization [18]. Moreover, our conclusions disagree with three reviews that reported that manipulation and mobilization [16, 19] or manipulation [20] is effective for subacromial impingement syndrome.

For ankle sprains, previous systematic reviews reported inconsistent results on the effectiveness of manual therapy [13–15]. Our findings on the effectiveness of manual therapy for the management of ankle sprains partially agree with two previous systematic reviews [13, 14], but disagree with one [15]. We found that mobilization provides only short-term improvements in activities and function and no clinically meaningful reduction in pain. Brantingham et al. and Loudon et al. concluded that manipulation and mobilization [13] or mobilization [14] provides short- and long-term benefits, including pain reduction [14]. Terada et al. concluded that mobilization is not effective [15].

The diverging conclusions between our review and previous systematic reviews can be attributed to differences in methodology and the publication of new evidence [13–21]. The conclusions of previous reviews may have been affected by the inclusion of studies that included manual therapy as a component of a multimodal program of care [13, 16, 18]. It is not possible to determine the specific effect of a modality when included in a multimodal program of care; the effectiveness of manual therapy may not be isolated from the effects of the other interventions in the multimodal program of care. Second, all [13–17, 19–21] but one previous review [21] included small trials which are more likely to suffer from Type II error and residual confounding. Third, one systematic review may have used a different search strategy and may have missed relevant studies [15]. Finally, all previous systematic reviews used a cut-off score to determine the internal validity of RCTs (using a checklist to critically appraise studies) [13–21]. This may limit the ability to appraise the impact of bias on study results.

### Recommendations for future studies

Our systematic review demonstrates that there is a lack of high-quality RCTs to inform the effectiveness of manual therapy for the management of recent and persistent MSDs of the upper and lower extremities. Our systematic review identified seven relevant RCTs. Four of them had major methodological issues and biases (i.e., unclear randomization and concealment procedure, inappropriate blinding, imbalanced baseline characteristics, invalid and unreliable outcome measures, high attrition rate) that markedly compromised their internal validity. Furthermore, only shoulder and ankle MSDs in adults were investigated by the three high-quality studies. In consideration of the noted prevalence and burden of MSDs of the upper and lower extremities, future studies should use rigorous methodology and focus on common MSDs of the extremities in both adults and children.

### Strengths and limitations

Our review has strengths. First, we implemented a comprehensive and rigorous search strategy that was checked through peer review. Second, we defined explicit inclusion and exclusion criteria to identify all possibly relevant studies. Third, we utilized two independent reviewers for screening and critical appraisal to minimize error and bias. Our methodology was standardized, and all reviewers were trained in critical appraisal prior to commencing the systematic review. Fourth, the SIGN criteria were utilized to standardize the critical appraisal process and to inform our scientific judgment. Lastly, we conducted best-evidence syntheses, excluding studies of low quality to minimize the risk of bias.

Our review also has limitations. First, we limited our search to studies published in the English language, which may have excluded some relevant studies. However, this is an unlikely source of bias as the majority of trials are published in English. The restriction of systematic reviews to the English language has not led to biased results in previous publications [60–62, 63, 64]. Second, our search strategy may have missed potentially relevant studies despite our broad definition of MSDs of the upper and lower extremities. Third, our review may have missed potentially relevant studies published prior to 1990. Finally, the critical appraisal process entails scientific judgment that may differ between reviewers. This potential bias was minimized by training reviewers on the use of a standardized critical appraisal tool and making an overall informed decision.

## Conclusion

The current evidence on the effectiveness of manual therapy for MSDs of the upper and lower extremities is limited. The available evidence supports the effectiveness of manual therapy in adults for the management of non-specific shoulder pain and grade I-II ankle sprains; however, it does not support the effectiveness of neck mobilization in adults for the management of subacromial impingement syndrome. We did not identify studies evaluating the effectiveness of manual therapy in children with MSDs of the upper and lower extremities.

## Appendix 1

### Ovid MELINE search strategy (upper extremity). Description data: Ovid MEDLINE search strategy for musculoskeletal disorders of the upper extremity

1. exp Upper Extremity/
2. Shoulder Pain/
3. exp "Sprains and Strains"/
4. exp Cumulative Trauma Disorders/
5. exp Median Neuropathy/
6. Shoulder Impingement Syndrome/
7. Shoulder Joint/in [Injuries]
8. Rotator Cuff/
9. Shoulder/in [Injuries]
10. exp Arm Injuries/
11. exp Hand Injuries/
12. Wrist Injuries/
13. Finger Injuries/
14. exp Tendinopathy/
15. Radial Neuropathy/
16. exp Ulnar Neuropathies/
17. exp Brachial Plexus/
18. Bursitis/
19. Thoracic Outlet Syndrome/
20. carpal tunnel syndrome.ab,ti.
21. (medial and (epicondylitis or epicondylosis or epicondylopathy)).ab,ti.
22. (lateral and (epicondylitis or epicondylosis or epicondylopathy)).ab,ti.
23. (shoulder* and (pain* or sprain* or strain* or injur* or impair* or impingement)).ab,ti.
24. (shoulder* and (tendinopathy or tendinitis or tendonitis or capsulitis)).ab,ti.
25. ((glenohumeral or scapul* or acromioclavicular) and (pain* or sprain* or strain* or injur*)).ab,ti.
26. (rotator cuff and (sprain* or strain* or tear* or bursitis tendinitis or impingement)).ab,ti.
27. ((supraspinatus or infraspinatus or subscapularis or teres minor or teres major or trapezius or deltoid or bicep* or bicipital or coracobrachialis) and (impingement or strain* or tear* or pain*)).ab,ti.
28. biceps tend?nitis.ab,ti.
29. painful arc.ab,ti.
30. frozen shoulder.ab,ti.
31. (shoulder and capsul* and (sprain* or tear*)).ab,ti.
32. (forearm* and (pain* or sprain* or strain* or injur* or impair*)).ab,ti.
33. (arm* and (pain* or sprain* or strain* or injur* or impair*)).ab,ti.
34. (wrist* and (pain* or sprain* or strain* or injur* or impair*)).ab,ti.
35. (hand* and (pain* or sprain* or strain* or injur* or impair*)).ab,ti.
36. (finger* and (pain* or sprain* or strain* or injur* or impair*)).ab,ti.
37. (elbow* and (pain* or sprain* or strain* or injur* or impair*)).ab,ti.
38. "thoracic outlet syndrome*".ab,ti.
39. tennis elbow.ab,ti.
40. peritendinitis.ab,ti.
41. (rotator cuff and (injur* or disorder*)).ab,ti.
42. (median adj neuropath*).ab,ti.
43. (radial adj neuropath*).ab,ti.
44. "De Quervain’s tenosynovit*".ab,ti.
45. brachial plexus.ab,ti.
46. bursitis.ab,ti.
47. "upper extremit* injur*".ab,ti.
48. ((radial or ulnar) adj neuropath*).ab,ti.
49. "cumulative trauma disorder*".ab,ti.
50. "cubital tunnel syndrome*".ab,ti.
51. "overuse syndrome*".ab,ti.
52. (repetit* and (strain* or sprain* or injur* or disorder*)).ab,ti.
53. or/1-52
54. Musculoskeletal Manipulations/
55. Manipulation, Spinal/
56. Manipulation, Chiropractic/
57. Manipulation, Orthopedic/
58. Manipulation, Osteopathic/
59. Motion Therapy, Continuous Passive/
60. Muscle Stretching Exercises/
61. (manipulat* adj4 (spinal or lumbar or thoracic or cervical)).ab,ti.
62. (mobili?ation adj4 (spinal or lumbar or thoracic or cervical)).ab,ti.
63. (manipulat* adj4 (chiropract* or osteopath* or orthopedic* or orthopaedic*)).ab,ti.
64. (mobili?ation adj4 (chiropract* or osteopath* or orthopedic* or orthopaedic*)).ab,ti.
65. (adjustment* adj4 (chiropract* or spinal or lumbar or cervical or thoracic)).ab,ti.
66. (therap* adj4 (manual or manipulat* or mobili?at*)).ab,ti.
67. (traction and (manual or passive or mechanical or non-surgical or nonsurgical)).ab,ti.
68. (flexion-distraction or flexion distraction).ab,ti.
69. (HVLA or high velocity low amplitude).ab,ti.
70. (manipulat* and (instrument assisted or instrument-assisted)).ab,ti.
71. (manipulat* and (physiotherap* or physical therap*)).ab,ti.
72. (mobili?ation and (physiotherap* or physical therap*)).ab,ti.
73. (musculoskeletal and (physiotherap* or physical therap*)).ab,ti.
74. or/54-73
75. Randomized Controlled Trials as Topic/
76. Controlled Clinical Trials as Topic/
77. Clinical Trials as Topic/
78. exp case–control studies/
79. exp Cohort Studies/
80. Double-Blind Method/
81. Single-Blind Method/
82. Placebos/
83. randomized controlled trial.pt.
84. controlled clinical trial.pt.
85. comparative study.pt.
86. (meta analys* or meta-analys* or metaanalys*).ab,ti.
87. (cohort and (study or studies or analys*)).ab,ti.
88. (random* and (control* or clinical or allocat*)).ab,ti.
89. (case adj control*).ab,ti.
90. ((double or single) and blind*).ab,ti.
91. "placebo*".ab,ti.
92. (comparative and (study or studies)).ab,ti.
93. or/75-92
94. 53 and 74 and 93
95. limit 94 to (english language and humans and yr = "1990 - 2015")

## Appendix 2

### Ovid MEDLINE search strategy (lower extremity). Description data: Ovid MEDLINE search strategy for musculoskeletal disorders of the lower extremity

1. Randomized Controlled Trials as Topic/
2. Controlled Clinical Trials as Topic/
3. Clinical Trials as Topic/
4. exp Case–control Studies/
5. exp Cohort Studies/
6. Double-Blind Method/
7. Single-Blind Method/
8. Placebos/
9. randomized controlled trial.pt.
10. controlled clinical trial.pt.
11. comparative study.pt.
12. (meta analys* or meta-analys* or metaanalys*).ab,ti.
13. (cohort and (study or studies or analys*)).ab,ti.
14. (random* and (control* or clinical or allocat*)).ab,ti.
15. (case adj control*).ab,ti.
16. ((double or single) and blind*).ab,ti.
17. "placebo*".ab,ti.
18. (comparative and (study or studies)).ab,ti.
19. (meta analys* or meta-analys* or metaanalys*).ab,ti.
20. or/1-19
21. exp Lower Extremity/
22. exp Hip Injuries/
23. exp Leg Injuries/
24. exp Knee Injuries/
25. exp Foot/
26. exp Toes/in [Injuries]
27. exp Knee Joint/
28. exp Foot Bones/
29. Anterior Cruciate Ligament/
30. Posterior Cruciate Ligament/
31. exp Collateral Ligaments/
32. Ankle Injuries/
33. Ankle Joint/
34. Ankle/
35. Lateral Ligament, Ankle/in [Injuries]
36. Fasciitis, Plantar/
37. (lower and (extremit* or limb* or injur*)).ab,ti.
38. (ankle* and (sprain* or strain* or injur*)).ab,ti.
39. ((talofibular or calcaneofibular or calcaneotibial or tibio*) and (sprain* or strain* or injur*)).ab,ti.
40. (deltoid and ankle*).ab,ti.
41. (fibularis and strain*).ab,ti.
42. ((peroneal or peroneus) and strain*).ab,ti.
43. (tibialis and strain* and (anterior or posterior)).ab,ti.
44. (band syndrome and (illiotibial or IT)).ab,ti.
45. achilles.ab,ti.
46. (ACL or LCL or MCL or PCL).ab,ti.
47. "adductor muscle*".ab,ti.
48. "collateral ligament*".ab,ti.
49. gastrocnemius.ab,ti.
50. (gluteus or gluteal).ab,ti.
51. "hamstring*".ab,ti.
52. "hip flexor*".ab,ti.
53. "hoffa* syndrome".ab,ti.
54. iliofemoral.ab,ti.
55. impingement.ab,ti.
56. (buttock* and (injur* or pain*)).ab,ti.
57. (foot and (injur* or pain*)).ab,ti.
58. (hip* and (injur* or pain*)).ab,ti.
59. (knee* and (injur* or pain*)).ab,ti.
60. (leg* and (injur* or pain*)).ab,ti.
61. (thigh* and (injur* or pain*)).ab,ti.
62. (toe* and (injur* or pain* or turf)).ab,ti.
63. ischiofemoral.ab,ti.
64. "metatars*".ab,ti.
65. "patellofemoral pain syndrome*".ab,ti.
66. "patellar tendon*".ab,ti.
67. popliteus.ab,ti.
68. pubofemoral.ab,ti.
69. "quadricep*".ab,ti.
70. soleus.ab,ti.
71. talocrural.ab,ti.
72. "tarsal*".ab,ti.
73. tendinosis.ab,ti.
74. tendinopathy.ab,ti.
75. plantar fasciitis.ab,ti.
76. tibialis.ab,ti.
77. or/21-76
78. Musculoskeletal Manipulations/
79. Manipulation, Spinal/
80. Manipulation, Chiropractic/
81. Manipulation, Orthopedic/
82. Manipulation, Osteopathic/
83. Motion Therapy, Continuous Passive/
84. Muscle Stretching Exercises/
85. (manipulat* and (spinal or lumbar or thoracic or cervical)).ab,ti.
86. (mobili?ation and (spinal or lumbar or thoracic or cervical)).ab,ti.
87. (manipulat* and (chiropract* or osteopath* or orthopedic* or orthopaedic*)).ab,ti.
88. (mobli?ation and (chiropract* or osteopath* or orthopedic* or orthopaedic*)).ab,ti.
89. (adjustment* and (chiropract* or spinal or lumbar or cervical or thoracic)).ab,ti.
90. (therap* and (manual or manipulat* or mobili?at*)).ab,ti.
91. (traction and (manual or passive or mechanical or non-surgical or nonsurgical)).ab,ti.
92. (flexion-distraction or flexion distraction).ab,ti.
93. (HVLA or high velocity low amplitude).ab,ti.
94. (manipulat* and (instrument assisted or instrument-assisted)).ab,ti.
95. (manipulat* and (physiotherap* or physical therap*)).ab,ti.
96. (mobili?ation and (physiotherap* or physical therap*)).ab,ti.
97. (musculoskeletal and (physiotherap* or physical therap*)).ab,ti.
98. or/78-97
99. 20 and 77 and 98
100. limit 99 to (english language and humans and yr = "1990 -2015")

## Abbreviations

ADL: Activities of daily living
CCGPP: The Council for Chiropractic Guidelines and Practice Parameters
CDC: The Centers for Diseases Control and Prevention
CI: Confidence interval
FAAM: The Foot and Ankle Ability Measure
LEFS: The Lower Extremity Functional Scale
K: Kappa statistic
MCID: The minimal clinically important difference
MeSH: Medical Subject Headings
NRS: The Numeric Rating Scale
PRESS: The Peer Review of Electronic Search Strategies
PRISMA: Preferred Reporting Items for Systematic Reviews and Meta-Analyses
PROSPERO: The International Prospective Register of Systematic Reviews
QuickDASH: The Quick Disabilities of the Arm, Hand, and Shoulder
RCT: Randomized controlled trial
RR: Relative risk
SDQ: The Shoulder Disability Questionnaire
SIGN: The Scottish Intercollegiate Guidelines Network
WSIB: The Workplace Safety and Insurance Board
